# Diagnostic Accuracy of Microscopic Observation Drug Susceptibility (MODS) Assay for Pediatric Tuberculosis in Hanoi, Vietnam

**DOI:** 10.1371/journal.pone.0072100

**Published:** 2013-09-04

**Authors:** Sinh Thi Tran, John Patrick Renschler, Hai Thanh Le, Hang Thi Thu Dang, Tuan Minh Dao, An Nhat Pham, Liem Thanh Nguyen, Hung Van Nguyen, Thuy Thi Thu Nguyen, Sy Ngoc Le, Annette Fox, Maxine Caws, NhuDo Thi Quynh, Peter Horby, Heiman Wertheim

**Affiliations:** 1 National Hospital of Pediatrics, Hanoi, Vietnam; 2 National Lung Hospital, Hanoi, Vietnam; 3 Oxford University Clinical Research Unit, Hanoi, Vietnam; 4 Grand Health Challenge, Princeton Environmental Institute, Princeton University, Princeton, New Jersey, United States of America; 5 Centre for Tropical Medicine, Nuffield Department of Clinical Medicine, University of Oxford, Oxford, United Kingdom; 6 Oxford University Clinical Research Unit, Ho Chi Minh City, Vietnam; University of Melbourne, Royal Children's Hospital Melbourne, Australia

## Abstract

**Introduction:**

icroscopic Observation Drug Susceptibility **(**MODS) has been shown to be an effective and rapid technique for early diagnosis of tuberculosis (TB). Thus far only a limited number of studies evaluating MODS have been performed in children and in extra-pulmonary tuberculosis. This study aims to assess relative accuracy and time to positive culture of MODS for TB diagnosis in children admitted to a general pediatric hospital in Vietnam.

**Methods/Principal Findings:**

Specimens from children with suspected TB were tested by smear, MODS and Lowenstein-Jensen agar (LJ). 1129 samples from 705 children were analyzed, including sputum (n = 59), gastric aspirate (n = 775), CSF (n = 148), pleural fluid (n = 33), BAL (n = 41), tracheal fluid (n = 45), other (n = 28). 113 TB cases were defined based on the “clinical diagnosis” (confirmed and probable groups) as the reference standard, in which 26% (n = 30) were diagnosed as extra-pulmonary TB. Analysis by patient shows that the overall sensitivity and specificity of smear, LJ and MODS against “clinical diagnosis” was 8.8% and 100%, 38.9% and 100%, 46% and 99.5% respectively with MODS significantly more sensitive than LJ culture (P = 0.02). When analyzed by sample type, the sensitivity of MODS was significantly higher than LJ for gastric aspirates (P = 0.004). The time to detection was also significantly shorter for MODS than LJ (7 days versus 32 days, P<0.001).

**Conclusion:**

ODS is a sensitive and rapid culture technique for detecting TB in children. As MODS culture can be performed at a BSL2 facility and is inexpensive, it can therefore be recommended as a routine test for children with symptoms suggestive of TB in resource-limited settings.

## Introduction

In 2010 there were an estimated 1 million cases of pediatric tuberculosis (TB) and 130,000 associated deaths in the world [1]. The overall 13% global mortality rate is considerably higher than the 0.5% mortality rate in pediatric tuberculosis recorded in the United States and other developed nations [2]. This gap reflects the need for accelerated diagnosis and initiation of appropriate chemotherapy in order to improve outcomes of pediatric TB cases within developing nations. Underdiagnosis of TB is a critical concern as children are failing to receive appropriate treatment [3].

Currently, diagnosis is complicated by three factors. First, high rates of extra-pulmonary disease are responsible for non-specific clinical presentation of tuberculosis resulting in underdiagnosis [4]–[6]. Second, adequate bacteriologic specimens are difficult to obtain because children have difficulties in expectorating sputum for microbiologic testing. Finally, when samples are obtained, available tests are often insensitive, slow, or too expensive due to the low bacillary loads in specimens from children [7], [8]. As a result, children who lack typical symptoms are seldom referred to TB hospitals where they can receive additional testing and appropriate treatment.

In Vietnam and other high TB burden countries, Ziehl-Neelsen (ZN) staining of acid-fast bacilli (AFB) is the primary method of diagnosis. While results are typically available within hours of sample collection, the test relies on the experience of the microscopist and is best suited for detecting high concentrations of AFB that are uncommon in pediatric specimens [9]. In addition to poor sensitivity, the test cannot reveal drug resistance and cannot distinguish between mycobacterium species [5]. In Vietnam, referral TB hospitals have access to more sensitive culture-based techniques. The most commonly used solid culture method is culture on Lowestein-Jensen (LJ) media, which is more sensitive than ZN staining but requires long incubation times of up to 6 weeks [10]–[12]. Commercial liquid culture systems, such as BACTEC MGIT 960 (Becton Dickinson, USA) and MB/BacT (Biomerieux, France), significantly reduce the time to detection and have higher sensitivities than LJ culture (9–22 days), but the high costs prevent use in resource-constrained settings [10], [13].

The microscopic observation drug susceptibility (MODS) assay has been estimated to cost less than one dollar per test in 2007 and has been endorsed by WHO [14]. MODS is a closed liquid culture system and has a low risk of contamination. The test was recently validated in Ho Chi Minh City and Lima as a sensitive and rapid liquid culture technique for TB detection in pediatric specimens [15]–[17]. While these MODS pediatric studies produced promising results, they were performed in populations in whom TB was highly suspected with a high pre-test probability of TB. These evaluations in referral populations may overestimate the clinical utility of the assay in a general population where the primary pediatric TB diagnosis is usually made. In addition, there have been limited reports of the MODS assay for diagnosis of extra-pulmonary TB. To that end our study aimed to assess relative accuracy and time to positive culture of MODS for diagnosis of both pulmonary and extra-pulmonary TB in children admitted to a large general pediatric hospital in Vietnam.

## Materials and Methods

**Figure 1:**
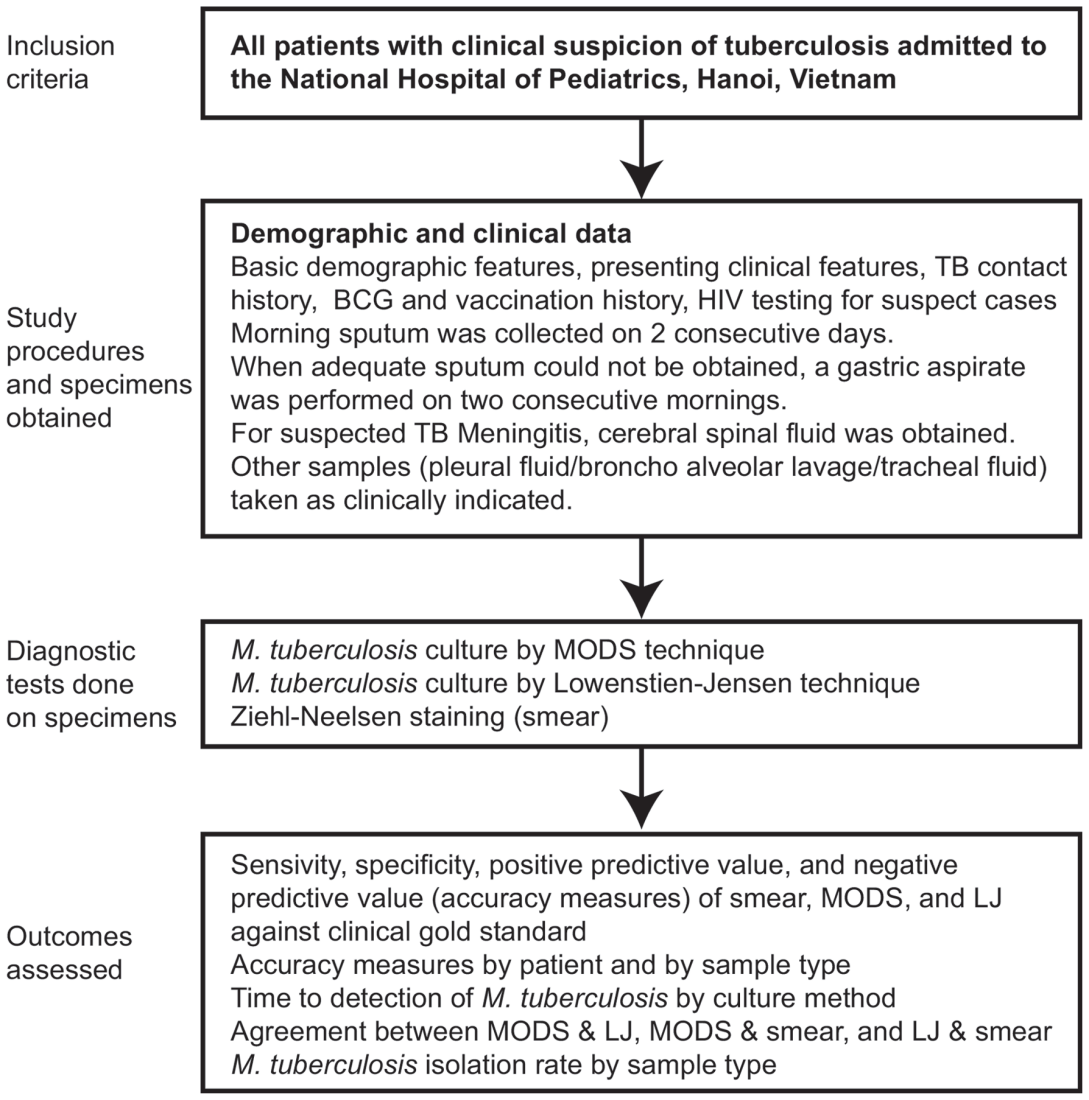
Study Design.

The study design is summarized in Figure 1. The National Hospital of Pediatrics (NHP) implemented MODS to detect TB in children as of February 2009. NHP is the largest hospital for pediatric care in northern Vietnam with 1,100 beds. Annually, approximately 60,000 patients are admitted to NHP. The MODS laboratory was established in a dedicated room of the general microbiology laboratory of NHP and was equipped with a biosafety cabinet, centrifuge, refrigerator, an inverse microscope and an incubator. Children with clinical suspicion of tuberculosis presenting at NHP, Vietnam were tested with ZN stain, MODS and LJ culture. In this study we evaluated MODS prospectively from February 2009 to December 2010.

Suspected TB cases were considered to have at least two of the following signs: unexplained fever for more than 1 week, unexplained cough for more than 1 week, radiographic findings suggestive of tuberculosis, failure to thrive or malnutrition or weight loss compared against the standard growth chart defined by WHO guidelines [18], enlarged non-tender lymph nodes or lymph node abscess, signs of meningitis with history of at least 1 week, HIV positive, contact with TB source within the preceding 2 years. Weight loss was defined as unexplained reduction in weight compared with the highest weight recorded in last 3 months. Failure to thrive or malnutrition was recorded if the weight for age or weight for height z score were equal or less than 2 in the normal growth chart. For HIV positive individuals, the definition included lack of response to nutritional rehabilitation or antiretroviral therapy. TB contact history was investigated by interviewing the patient or patient's parent. Any exposure to a case of tuberculosis was considered as having TB history contact. The presence of BCG scar on the arm of the patient was also checked by study clinicians. Data on demographic features, TB history, TB contact history, HIV status and presenting clinical feature were collected on a standard MODS test request form. HIV testing is not performed routinely at NHP and the decision to offer an HIV test is determined by the treating clinician. The treating clinicians collected samples according to routine protocol, depending on presenting symptoms.

Patients were categorized into one of three groups: confirmed TB, probable TB or TB unlikely based on microbiological findings, intention to treat and outcome. Confirmed TB cases had a positive LJ culture and clinical symptoms consistent with TB. Patient status was defined as “probable TB” if the patient had clinical symptoms consistent with TB, did not improve with standard antibiotic and was transferred to National Lung Hospital for treatment in accordance with routine hospital practice but lacked microbiological confirmation. In this group, standard clinical evaluation by the treating clinician determined the decision to treat for TB, including symptom evaluation, history and chest X-ray findings as appropriate to the form of suspected TB. The clinical assessment was made by the treating clinician and was not pre-defined by the study. Patient status was defined as “TB unlikely” if the patient had an alternative diagnosis and treatment. This study was designed before the recently published standardized case definition for research on childhood pulmonary TB [19].

### Ethics

This study was approved by the Institutional Review Board of NHP. The need for informed consent was waived by NHP ethics committee as the study was considered to be an evaluation of medical services and did not alter routine patient care at NHP.

### Sample collection

For pulmonary TB diagnosis, two gastric fluid aspirates or two sputum samples were collected on successive mornings for microbiological testing at NHP. In case of suspected TB meningitis (TBM), cerebrospinal fluid (CSF) was obtained with a recommended minimum volume of one milliliter. In case of other presenting symptoms, other samples (broncho alveolar lavage, pleural fluid, biopsy, or others as appropriate) were taken as clinically indicated at the discretion of the treating clinician. All specimen types referred for TB culture were assessed in this study. For each sample, one aliquot was reserved for smear and MODS culture at NHP, and another aliquot was transferred to National Lung Hospital (NLH) for LJ culture. Morning specimens were processed for both culture methods on the same day of collection; specimens collected in different time in day were stored overnight at 4°C and cultured on the following day.

### Sample processing

All samples, except for CSF, were homogenized and decontaminated with NaOH-NALC 2%. In brief, the sample aliquot at NHP was added to an equal volume of homogenization buffer and decontamination buffer in a 15 ml falcon tube. The tube was lightly shaken by an automated shaker and left at room temperature for 20 minutes. After that, an equal volume of phosphate buffer 1X was added to the mixture. The mixture was then vortexed and centrifuged at 3000 g, 4°C for 15 minutes. The supernatant was discarded and the pellet was re-suspended in 1 ml PBS. This suspension was then aliquoted into two eppendorfs, one for smear and one for MODS culture.

### TB smear

Two drops of pellet from each sample were put onto a slide for homogenous smear preparation. The smears were then stained by Ziehl Neelsen (ZN) method according to World Health Organization (WHO) standard protocol [20].

### Lowenstein-Jensen culture

For culture at the National Lung Hospital, after decontamination, a drop of sediment was placed onto Löwenstein-Jensen (LJ) medium prepared according to international standards. LJ cultures were examined visually weekly for up to 8 weeks. Final identification of the isolates was performed according to standard recommendations.

### MODS culture

The MODS method was performed as described by Caws et al [14]. Briefly, MODS media was prepared with 5.9 g Middlebrook 7H9 broth (Difco, USA), 3.1 ml glycerol and 1.25 g bacto casitone (Difco, USA) in 880 ml of sterile distilled water. The medium was autoclaved and stored in 22 ml aliquots at 4°C. We incubated one aliquot of each new batch of medium at 37°C for one week to check the sterility. Oleate-albumin-dextrose-catalase (OADC) and PANTA antibiotic supplement (Becton Dickinson, USA) were added into each tube immediately before use as MODS medium to reach final concentrations of 5.5% and 0.22% respectively. Culture was set up in 48-well MODS plate every day, except weekends. Each well contained 750 ml MODS medium and 250 ml processed sample. One positive control (BCG strain) and one negative control well (sterile distilled water) were added to each plate. To reduce the risk of cross-contamination, samples were added to alternating wells. The plate was incubated at 37°C, and the evidence of bacterium growth was recorded every other day after an initial five days of incubation. The sample was recorded as positive if any cord formation was seen. Likewise if no cord formation was seen after 3 months, the sample was recorded as negative. Any growth or turbidity in the negative control well was recorded as contamination.

### Contamination analysis

All MODS positive cultures were typed by 15-loci MIRU typing [21] to detect potential cross-contamination. The positive control culture inoculated to each MODS plate was a BCG strain to enable easy detection of cross-contamination from the positive control. Furthermore, we left negative control (non-inoculated) wells in between inoculated wells to check for contamination.

### Statistical Methods

Accuracy measures of smear, LJ and MODS culture were calculated on clinical diagnosis (clinical reference standard including confirmed TB group and probable TB group) as the reference test. In addition, we analyzed data on a ‘per patient’ and ‘per sample’ basis. In per patient analysis, the patient was regarded as positive if at least one sample yielded a positive test result. Exact binomial confidence intervals for accuracy measures (sensitivities, specificities, positive and negative prediction values) were calculated with the epiR package for R [22]. Comparisons of accuracies between tests were done using an Exact McNemar's test. For the time to detection analysis, we performed a log rank test on the ‘days to positive culture’ data for samples that were both LJ and MODS positive [23].

Demographic and clinical features of patients between all TB groups (confirmed, probable and unlikely) were compared using a chi-squared test for categorical data and Kruskal-Wallis test for continuous data. When comparing only 2 groups at a time, Fisher's exact test was used for categorical data, and a Wilcoxon rank sum test for continuous data.

All reported confidence intervals are two-sided 95% confidence intervals and p-values≤0.05 were regarded as statistically significant. All analyses were done with R program [24].

## Results

From 1^st^ February 2009 to 31^st^ December 2010, 726 children suspected of having TB were evaluated in this study. Twenty-one patients were excluded from the analysis because there was insufficient specimen collected to be able to perform LJ culture. 1129 samples from 705 children remained: sputum (n = 59), gastric aspirate (n = 775), CSF (n = 148), pleural fluid (n = 33), BAL (n = 45), tracheal fluid (n = 41), other (n = 28). The other samples included nasal washes (n = 2), fine needle lymph node aspirates (n = 7), pericardial fluid (n = 1), nasal pharyngeal aspirates (n = 8), peritonitis aspirate (n = 1) and pus in the joint (n = 1).

**Figure 2:**
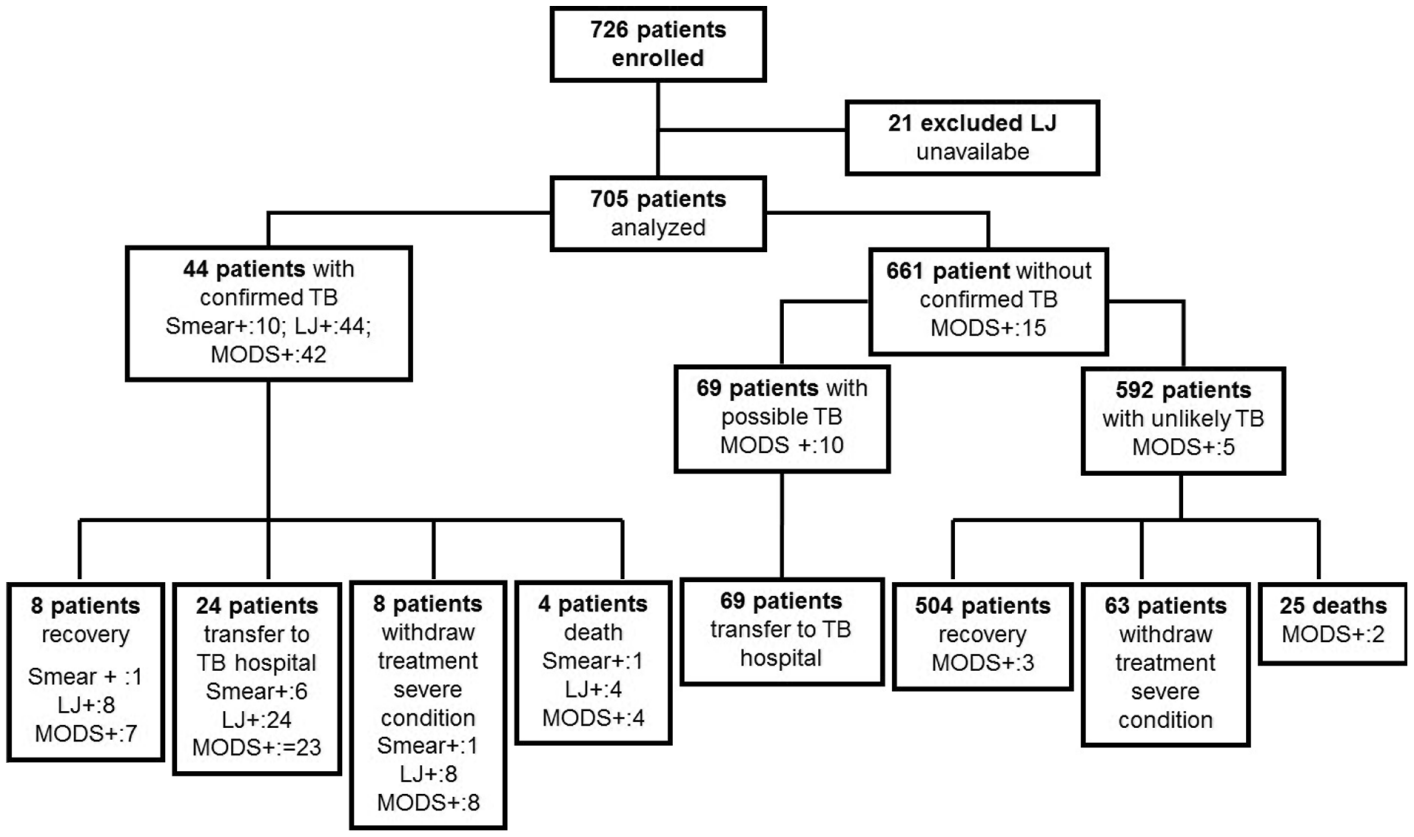
Patient Recruitment and Assignment to ‘confirmed TB’, ‘probable TB’, or ‘TB unlikely’ group.

Forty-four patients (6.2%, n = 44/705) had microbiological confirmation by LJ culture (‘confirmed TB’). Sixty-nine (9.8%, n = 69/705) patients were classified as ‘TB probable’ (see figure 2). Among unlikely TB patients (n = 561), 98.5% of patients (n = 552) were diagnosed as having one of the following infectious disease syndromes: respiratory, non-TB (68.8%), central nervous system diseases (17.1%) or prolonged fever of unknown origin (12.7%).

### Demographic Characteristics

General demographic characteristics of the study population are presented in Table 1. In brief, the male to female ratio was 1.9∶1 and the median age was 2 months (IQR  = 1–48 months). The confirmed and probable TB cases were significantly older than the unlikely TB group (P = 0.016, P = 0.001, respectively). Only 55.7% had evidence of BCG vaccination. 9.2% (n = 65/344 tested) of patients were HIV positive, and the confirmed and probable groups had significantly higher proportions of HIV positive children than the unlikely group: 18.2%, 15.9% and 7.7%, respectively (P<0.05). History taking showed that 6.5% of the children had a TB contact and 80.4% of those reported contacts were with a household family member. The confirmed and probable groups had a significantly higher proportion of reported TB contacts than the unlikely group: 15.9%, 17.4% and 4.6%, respectively (P<0.05).

**Table 1:** Demographic characteristics of patients.

Category	Subcategory	Total Population N = 705	Confirmed N = 44	Probable N = 69	Unlikely N = 592	Comparison*
Age	Median (IQR) *in months*	2 (1–48)	23 (2–87)	36 (1–96)	2 (1–42)	P<0.001 [P1 = 0.995; P2 = 0.001; P3 = 0.003]
	<5 years	545 (77.3)	29 (65.9)	41 (59.4)	475 (80.2)	P<0.001 [P1 = 0.544; P2<0.001; P3 = 0.033]
Sex	Male	463 (65.7)	22 (50)	48 (69.6)	391 (66)	P = 0.073
BCG Vaccination	Yes	393 (55.7)	26 (59.1)	39 (56.5)	328 (55.4)	..
	No	44 (6.2)	5 (11.3)	5 (7.2)	34 (5.7)	P = 0.310
	Unknown	268 (38)	13 (29.5)	25 (36.2)	230 (38.9)	..
HIV Status	Positive	65 (9.2)	8 (18.2)	11 (15.9)	46 (7.7)	P = 0.009 [P1 = 0.8; P2 = 0.037; P3 = 0.025]
	Negative	279 (39.6)	17 (38.6)	36 (52.2)	226 (38.2)	..
	Unknown	361 (51.2)	19 (43.2)	22 (31.9)	320 (54.1)	..
TB Contact	Yes	46 (6.5)	7 (15.9)	12 (17.4)	27 (4.6)	P<0.001 [P1 = 1; P2<0.001; P3 = 0.006]
	Yes & Contact was household family member	37 (80.4)	7 (100)	11 (91.7)	19 (70.1)	P = 0.111
	No	440 (62.4)	28 (63.6)	35 (50.7)	377 (63.7)	..
	Unknown	219 (31.1)	9 (20.5)	22 (31.9)	188 (31.8)	..

Summary measure for categorical data is n (%). * P is used to compare across all three groups (confirmed, probable, and unlikely). For continuous variables a Kruskal-Wallis rank sum test was used. When P<0.05 Fisher's Exact test was used to compare confirmed with probable (P1), probable with unlikely (P2), and confirmed with unlikely (P3). For continuous variables a Wilcoxon rank sum test was used for P1, P2, and P3 calculations.

### Clinical Symptoms

Clinical records could not be obtained for 115 patients and therefore the data available only concerns 590 of the 705 study patients. Clinical symptoms of TB of this referral study population (Table 2) included cough (73.7%), fever (80.2%), weight loss (22.5%), lymphadenopathy (11.9%), malnutrition (31.5%) and chest X-ray consistent with TB (26.4%). The median history of illness (14 days, IQR  = 8–20 days) did not vary significantly across groups. The confirmed TB group had a significantly higher proportion of patients with malnutrition (56.1%), weight loss (43.9%) and meningitis (51.2%) compared to the probable TB and unlikely TB groups (Table 2). The unlikely TB group had a significantly lower proportion of patients with chest X-ray suspected of TB than the confirmed TB and probable TB groups (21.8% versus 43.9% and 51.6%). Prevalence of lymphadenopathy was 22% amongst confirmed TB patients, 17.7% amongst probable TB patients, and 10.3% amongst unlikely TB patients. The difference between confirmed and unlikely groups was statistically significant (P = 0.035).

**Table 2:** Clinical features of 590 pediatric TB suspects.

Characteristic	Total Population N = 590	Confirmed N = 41	Probable N = 62	Unlikely N = 487	Comparison*
Cough	435 (73.7)	32 (78.0)	48 (77.4)	355 (72.9)	P = 0.6048
Fever	473 (80.2)	37 (90.2)	52 (83.9)	384 (78.9)	P = 0.158
Malnutrition	186 (31.5)	23 (56.1)	17 (27.4)	146 (30.0)	P = 0.001 [P1 = 0.003; P2 = 0.117; P3 = 0.001]
Weightloss	133 (22.5)	18 (43.9)	10 (16.1)	105 (21.6)	P = 0.002 [P1 = 0.003; P2 = 0.41; P3 = 0.003]
Lymphadenopathy	70 (11.9)	9 (22.0)	11 (17.7)	50 (10.3)	P = 0.027 [P1 = 0.619; P2 = 0.086; P3 = 0.035]
Meningitis	137 (23.2)	21 (51.2)	12 (19.3)	104 (21.4)	P<0.001 [P1 = 0.001; P2 = 0.869; P<0.001]
Chest X-ray suspected of TB	156 (26.4)	18 (43.9)	32 (51.6)	106 (21.8)	P<0.001 [P1 = 0.546; P2<0.001; P3 = 0.003]
History of Illness: median (IQR) *in days*	14 (8 – 20)	14 (8 – 20)	15 (10 – 25)	14 (8 – 24)	P = 0.639

Summary measure for categorical data is n (%).

* P is used to compare across all three groups (confirmed, probable, and unlikely). For continuous variables a Kruskal-Wallis rank sum test was used. When P<0.05 Fisher's Exact test was used to compare confirmed with probable (P1) , probable with unlikely (P2), and confirmed with unlikely (P3).

### Diagnostic Accuracy

The clinical reference standard was defined as patients who satisfied characteristics of either “confirmed TB” group or “probable TB” group. 113 patients (16%) were diagnosed with TB by clinical features and/or microbiological confirmation and 592 patients (84%) were classified as ‘TB unlikely’. See Table S1 for the diagnostic yield of three methods by disease spectrum in each patient group.

The sensitivity of MODS versus smear and LJ culture is summarized in Table 3. When analyzed by patient or specimen, MODS was more sensitive than smear (P<0.001 for both) and more sensitive than LJ culture (P = 0.019, P = 0.015). Specificity and positive predictive value (PPV) of MODS were 99.2% [95%CI: 98.0, 99.7] and 91.2% [95%CI: 80.7, 97.1] respectively. The negative predictive value (NPV) of smear, LJ culture, and MODS was 85.2% [95%CI: 82.3, 87.7], 90% [95%CI: 87, 91.8], and 90.6% [95%CI: 88.1, 92.7].

**Table 3:** Sensitivity of MODS, LJ culture, and smear against clinical reference standard.

	Sensitivity n (%) [95% CI]			P-Value*	
	MODS	LJ	Smear	vs LJ	vs Smear
**By patient (N = 113)**	52 (46) [36.6, 55.6]	44 (38.9) [29.9, 48.6]	10 (8.8) [4.3, 15.7]	0.019	<0.001
**By all samples (N = 195)**	69 (35.3) [28.7, 42.5]	59 (30.3) [23.9, 37.2]	11 (5.6) [2.8, 9.8]	0.015	<0.001
**By sample type:**					
Sputum (N = 23)	8 (34.8) [16.4, 57.3]	7 (30.4) [13.2, 52.9]	1 (4.3) [0.1, 21.9]	0.500	0.008
Gastric aspirate (N = 103)	28 (27.2) [19, 36.8]	20 (19.4) [12.3, 28.4]	3 (2.9) [0.6, 8.3]	0.004	<0.001
CSF (N = 33)	22 (66.6) [48.1, 82]	22 (66.6) [48.1, 82]	3 (9.1) [1.9, 24.3]	0.688	<0.001

The sensitivities of MODS, LJ culture, and smear were calculated against the clinical reference standard. Sensitivities were calculated by all samples (some patients provided more than one sample), by patient, and by sample type. Exact binomial 95% confidence intervals were calculated.

* Comparison of sensitivities using Exact McNemar's Test: MODS vs LJ and MODS vs Smear.

To investigate whether sample type had a strong impact on the sensitivity of the three methods, we investigated the number of each sample type collected from 113 clinically diagnosed TB patients and analyzed the sensitivity of these methods in terms of sputum sample, gastric aspirate, and CSF. Our data in Table 3 show that MODS was more sensitive than smear in all sample types, and more sensitive than LJ culture only for gastric aspirates. Table S2 shows the diagnostic yield of three methods by sample type.

Of the 79 samples that were MODS positive only, five were collected from five patients in the “unlikely TB” group. The LJ culture and molecular typing on these isolates confirm four cases of *M.tuberculosis* and one atypical mycobacterium. Inspection of inoculate position on culture plate and genotyping with MIRU-VNTR method ruled out the possibility of cross-contamination. Three of these five patients did not have enough remaining specimen for reculture and the remaining two were discharged to die at home. If the additional four cases detected by MODS are considered to be true TB cases, the adjusted sensitivity “by patient analysis” of smear, LJ, and MODS are 8.8% [95%CI: 4.3, 15.7], 38.9% [95%CI: 29.9, 48.6], and 49.6% [95%CI: 40.0, 59.0] respectively.

### Time to Detection

**Figure 3:**
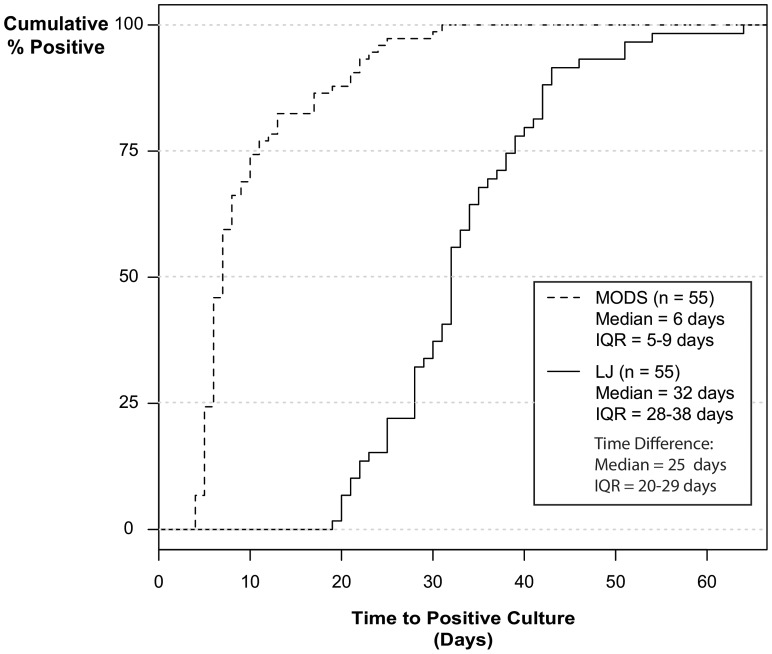
Paired Time to Detection by MODS and LJ Culture.

Time to detection was defined as the number of days from sample processing (day 1) to when results were available. Smear results were available on the same day as processing. Median time to detection using MODS was faster than LJ culture, 7 days (IQR: 6–11) vs. 35 days (IQR: 32–40). Six among seventy four MODS positive samples (8%) were detected after three weeks. In the 55 samples that were positive by both MODS and LJ culture, MODS was faster in all 55 samples (100%) and the median time difference was 25 (20–29) days in favor of MODS (log rank P<0.001) (see Figure 3).

### Contamination

The MODS assay contamination rate was low (26/1129, 2.3% of wells), and most contamination was due to fungi in samples from HIV patients. Cross contamination was not observed during this study period.

## Discussion

This study presents a direct comparison between MODS culture, LJ culture, and smear, across multiple specimen types, for diagnosis of pediatric pulmonary and extra-pulmonary tuberculosis in a large Vietnamese general pediatric hospital. MODS proved to be a relatively sensitive and rapid technique for the diagnosis of pediatric TB. MODS was significantly more sensitive than both smear (46% vs. 8.8%) and LJ (46% vs. 38.9%), and significantly faster than LJ with a median time difference of 25 days in favor of MODS. The sensitivity of MODS (46%) was slightly higher than the sensitivity reported in a pediatric study (39.7%) performed in HCMC, Vietnam [16]. For smear negative patients in our study, the sensitivity of LJ and MODS was 33% [95%CI: 24.1, 43] and 41.7% [95%CI: 32.1, 51.9] respectively. Most of the improved yield from MODS as compared to LJ in our study can be attributed to an improved yield from gastric aspirates where MODS sensitivity was 27.2% and LJ sensitivity was 19.4% (Exact McNemar Test P = 0.004).

The time to detection was also significantly shorter for MODS than LJ. Recent proposed MODS standard operation procedure states that if no growth is observed after day 21, the result is recorded as negative [25]. However, in this study 6 specimens were positive after day 21. Therefore, based on the finding in this study, the authors recommend that MODS culture should be incubated for at least one month.

In comparison with the HCMC study that was performed at a TB referral hospital, our study had a higher *M.tuberculosis* recovery rate from CSF samples (33/148 = 22.3% vs. 4/30 = 13.3%), and therefore a higher detection rate of pediatric TB meningitis. Nine of the 33 patients with positive CSF samples also had respiratory specimens positive for *M.tuberculosis*. It is possible the increased recovery rate from CSF samples in NHP could be attributed to difference in disease spectrum and empirical anti-tuberculosis treatment of patients with TBM prior to referral.

Furthermore, the participants in our study were much younger than those in the HCMC study. The mean (IQR) age of participants in the HCMC study was 9 years (3–13 years) whereas the mean (IQR) age of TB confirmed patients in our study was 24 months (10–87 months) [5]. Our study results therefore show that MODS has diagnostic utility in very young children [7].

This study has several limitations. Firstly, several patients were lost to follow-up at the TB hospital and their classification in the “probable TB” group may be a misclassification which would overestimate the specificity of the MODS assay. Patients may have been misclassified as probable TB when in fact they did not have TB due to a number of reasons. Once a patient has begun TB treatment this misclassification is difficult to detect. Treatment response is often not a reliable indicator of TB disease as patients may have undiagnosed drug resistance, deteriorate despite adequate therapy, or conversely, have an alternative bacterial infection which responds to antituberculous drugs or self-resolving viral illness and therefore improve on TB treatment without having TB.The impossibility of definitively categorizing cases is a recognized limitation in all studies assessing TB diagnostics in children [26]. The use of ‘intention to treat’ rather than a strictly defined case definition for probable TB was chosen to represent the spectrum of disease in a high-burden clinical setting. A strictly defined case criteria will likely over-estimate the sensitivity of a diagnostic test for TB (due to some true TB cases being misclassified as ‘not TB’), while a broad case definition (as used here) will usually under-estimate the sensitivity due to some non-TB cases being classified as TB. Although the MODS assay can be used to test for drug resistance, drug resistance testing (DST) was not performed by MODS in this study due to the paucibacillary nature of pediatric samples. Further study is needed to confirm if MODS direct DST is applicable for pediatric specimens.

The GeneXpert system (Cepheid, Belgium) is being implemented with rapid scale-up in developing nations as part of the WHO monitored global rollout plan [27]. The GeneXpert MTB/RIF assay is more sensitive than smear microscopy, performed in under two hours, safe for technicians, and accurate for detecting rifampicin resistance [28]. A cost-effectiveness analysis of TB diagnosis demonstrated that MTB/RIF use for initial diagnosis of suspected TB cases would substantially decrease TB associated mortality and morbidity at a reasonable cost, according to conventional cost-effectiveness benchmarks [29]. However, the accompanying sensitivity analysis shows that higher usage of culture methods would result in a less favorable cost-effectiveness ratio for GeneXpert MTB/RIF. GeneXpert MTB/RIF should not be considered a replacement for culture methods, especially considering the need to test for resistance beyond rifampicin [30]. Moreover, in regards to pediatric TB, a study of 452 suspected cases performed across two hospitals in Cape Town found that 25% of children with culture confirmed tuberculosis were negative by the GeneXpert MTB/RIF assay [31]. The implementation of the Xpert MTB/RIF test will increase case detection and considerably shorten time to treatment for those children who are diagnosed, but given the high rate of false negatives and a negotiated cartridge price of $10 USD/test, GeneXpert MTB/RIF alone will not be the solution for pediatric TB diagnosis in resource constrained settings [32].

There was one misclassification of NTM as *M.tuberculosis* in this study (1/69 positive MODS samples) due to cording observed in the MODS well. This issue has been addressed by a revision to the MODS standard protocol that includes additional wells containing p-nitrobenzoic acid (PNB) to distinguish *M.tuberculosis* from NTM growth [25].

In conclusion, MODS is a rapid low-cost diagnostic tool for TB diagnosis in the pediatric population. General pediatric hospitals in TB endemic countries can improve their TB diagnosis by implementing MODS in case other liquid culture systems are absent. Access to the MODS assay increases TB case-finding and early diagnosis which can shorten the time to appropriate therapy.

## Supporting Information

Table S1 **Tuberculosis diagnostic yield by spectrum disease among 705 children at NHP during 2009–2010 (separate file).**
(DOCX)

Table S2 **TB diagnostic yield by specimen type in 1129 samples collected from 705 pediatric patients admitted to NHP (separate file).**
(DOCX)
