# Correction: Diagnostic Accuracy of Microscopic Observation Drug Susceptibility (MODS) Assay for Pediatric Tuberculosis in Hanoi, Vietnam

**DOI:** 10.1371/annotation/ae927665-fe45-4126-b35d-f44576efab91

**Published:** 2013-09-20

**Authors:** Sinh Thi Tran, John Patrick Renschler, Hai Thanh Le, Hang Thi Thu Dang, Tuan Minh Dao, An Nhat Pham, Liem Thanh Nguyen, Hung Van Nguyen, Thuy Thi Thu Nguyen, Sy Ngoc Le, Annette Fox, Maxine Caws, Nhu Thi Quynh Nguyen, Peter Horby, Heiman Wertheim

There were errors in the name of the thirteenth author. The correct name is: Nhu Thi Quynh Nguyen.

On two occasions in the Abstract, the letter "M" was dropped from words.

"*Introduction:* icroscopic Observation Drug Susceptibility (MODS)..." should be "*Introduction:* Microscopic Observation Drug Susceptibility (MODS)..."

"*Conclusion:* ODS is a sensitive..." should be corrected as "*Conclusion:* MODS is a sensitive..." 

