# Supplementary material for: Diagnostic Accuracy of Microscopic Observation Drug Susceptibility (MODS) Assay for Pediatric Tuberculosis in Hanoi, Vietnam
Source: PLoS One. 2013 Sep 4;8(9):e72100. doi: 10.1371/journal.pone.0072100 (PMC3762843; doi:10.1371/journal.pone.0072100)
Supplement: Table S1 — Tuberculosis diagnostic yield by spectrum disease among 705 children at NHP during 2009–2010 (separate file). (DOCX) [file pone.0072100.s001.docx]

**Table S1: Tuberculosis diagnostic yield by spectrum disease among 705 children at NHP during 2009-2010**

| **Group** | **Diagnosis** | **AFB (n, %)** | **LJ (n, %)** | **MODS (n, %)** |
| --- | --- | --- | --- | --- |
| **Confirmed TB (n=44)** | PTB (n=22) | 6 (27.3) | 22 (100) | 21 (95.5) |
|  | TBM (n=21) | 4 (19.0) | 21 (100) | 20 (95.2) |
|  | Others (n=1) | 0 (0) | 1 (100) | 1 (100) |
| **Probable TB**  **(n=69)** | PTB (n=52) | 0 (0) | 0 (0) | 7 (13.5) |
|  | TBM (n=11) | 0 (0) | 0 (0) | 3 (27.3) |
|  | Others (n=6) | 0 (0) | 0 (0) | 0 (0) |
| **Not TB**  **(n=592)** | Suspected PTB (n=407) | 0 (0) | 0 (0) | 3 (0.7) |
|  | Suspected TBM (n=101) | 0 (0) | 0 (0) | 0 (0) |
|  | Others (n=84) | 0 (0) | 0 (0) | 1 (1.2) |

*n : number of MTB positive cases; %: percent of positive cases in the diagnosis group*

*AFB: Acid Fast Bacilli staining; LJ: Lowestein-Jensen culture;MODS: Microscopic Observation Drug Susceptibility assay*

*PTB: Pulmonary tuberculosis; TBM: Tuberculous meningitis*
