# Supplementary material for: Diagnostic Accuracy of Microscopic Observation Drug Susceptibility (MODS) Assay for Pediatric Tuberculosis in Hanoi, Vietnam
Source: PLoS One. 2013 Sep 4;8(9):e72100. doi: 10.1371/journal.pone.0072100 (PMC3762843; doi:10.1371/journal.pone.0072100)
Supplement: Table S2 — TB diagnostic yield by specimen type in 1129 samples collected from 705 pediatric patients admitted to NHP (separate file). (DOCX) [file pone.0072100.s002.docx]

**Table S2: TB diagnostic yield by specimen type in 1129 samples collected from 705 pediatric patients admitted to NHP during 2009-2010.**

|  | **Number of positive samples (n)** | | |
| --- | --- | --- | --- |
| **Specimen type** | **AFB** | **LJ** | **MODS** |
| Gastric aspirate (n=775) | 3 | 20 | 31 |
| Tracheal aspirate (n=41) | 0 | 2 | 2 |
| Pleural fluid (n=33) | 0 | 0 | 1 |
| BAL (n=45) | 3 | 6 | 5 |
| Sputum (n=59) | 1 | 7 | 8 |
| CSF (n=148) | 3 | 22 | 24 |
| Nasal wash (n=1) | 0 | 0 | 1 |
| Lymphnode aspirate (n=7) | 1 | 1 | 1 |
| Joint pus (n=1) | 0 | 1 | 1 |
| Others (19) | 0 | 0 | 0 |
| All (n=1129) | 11 | 59 | 74 |

*AFB: Acid Fast Bacilli Staining; LJ: Loweinstein Jensen culture; MODS: Microscopic Observation Drug Susceptibility assay; BAL: bronchoalveolar lavage*
